# Are eviscerations preventable?

**DOI:** 10.1590/0100-6991e-20253814-en

**Published:** 2025-02-13

**Authors:** JULIO FERNANDES TOMASI, FABIELLE MENEZES TOLFO, LAIS MADEIRA CONSTANTINO, FELIPE ANTÔNIO CACCIATORI

**Affiliations:** 1 - Hospital São José, Serviço de Cirurgia Geral - Criciúma - SC - Brasil; 2 - Universidade do Extremo Sul Catarinense - UNESC, Curso de Medicina - Criciúma - SC - Brasil

**Keywords:** Surgical Wound, Hernia, Abdominal, Abdominal Wall, Evisceration, Prophylactic Mesh, Deiscência da Ferida Operatória, Hérnia Incisional, Parede Abdominal, Evisceração, Tela Profilática

## Abstract

**Introduction:**

The incidence of eviscerations is 3.5% in the literature. The use of prophylactic meshes in patients at high risk of evisceration has been studied. The objective of this study is to evaluate the characteristics of patients undergoing abdominal wall resuturing due to evisceration and verify the benefit of using prophylactic mesh in this sample.

**Methods:**

This is a retrospective cohort study, which analyzed the medical records of patients who underwent abdominal wall resuturing procedures between January 2010 and December 2023 in a tertiary hospital. The inclusion criteria were patients who underwent abdominal wall resuturing in the study hospital, with index surgery in the same hospital and median access. Patients under 18 years of age, patients undergoing laparoscopic surgery and non-median access were excluded. The Rotterdam risk score for aponeurosis dehiscence, modified by Lima, was used as a parameter.

**Results:**

The final sample of 252 patients was made up of 74.2% men. The median age was 64 years and the median BMI was 24.3kg/m^2^. The median number of days between surgery and resuturing was 8. The median hemoglobin was 11.1g/dL. The incidence of neoplasia, smoking and COPD was 47.2%, 32.1% and 13% respectively. Elective surgeries were 58.8%.

**Conclusion:**

It was concluded that, using the modified Rotterdam score, of the 227 patients, 164 (72.2%) would have received prophylactic mesh, which potentially would have prevented evisceration.

## RESUMO

**Introdução:**

A incidência de eviscerações é de 3,5% na literatura. O uso de telas profiláticas em pacientes com alto risco de evisceração tem sido estudado. O objetivo deste estudo é avaliar as características dos pacientes submetidos à ressutura da parede abdominal devido evisceração e verificar o benefício do uso de tela profilática nesta amostra.

**Métodos:**

Trata-se de estudo retrospectivo do tipo coorte, que analisou os prontuários de pacientes submetidos ao procedimento de ressutura de parede abdominal entre janeiro de 2010 e dezembro de 2023 em um hospital terciário. Os critérios de inclusão foram pacientes submetidos à ressutura de parede abdominal no hospital de estudo, com cirurgia índex no mesmo hospital e acesso mediano. Pacientes menores de 18 anos, pacientes submetidos a cirurgias videolaparoscópicas e acessos não medianos foram excluídos. O escore de risco para deiscência de aponeurose de Rotterdam, modificado por Lima, foi utilizado como parâmetro.

**Resultados:**

A amostra final de 252 pacientes foi composta por 74,2% de homens. A mediana de idade foi de 64 anos e a mediana de IMC foi de 24,3kg/m^2^. A mediana do intervalo de dias entre a cirurgia e a ressutura foi de 8 dias. A hemoglobina mediana foi de 11,1g/dL. A prevalência, na amostra, de neoplasia, tabagismo e DPOC foi de 47,2%, 32,1% e 13%, respectivamente. Cirurgias eletivas foram 58,8%.

**Conclusão:**

Concluiu-se que, utilizando o escore de Rotterdam modificado, dos 227 pacientes, 164 (72,2%) teriam recebido tela profilática, o que, potencialmente, teria evitado a evisceração.

## INTRODUCTION

The median access, despite advances in minimally invasive techniques, is still widely used because it offers a view of the entire abdominal cavity^1^. A complication of this access in the postoperative period is the dehiscence of the surgical wound, which consists of partial or total rupture of its constituent planes. Evisceration is the rupture of all layers of the abdominal wall, with extrusion or exposure of the abdominal viscera^2^.

The incidence of evisceration is between 0.4% to 3.5%^3^ ^-^ ^5^, and most often occurs between the fourth and 14^th^ postoperative days^6^ ^,^ ^7^. In addition to evisceration, late incisional hernia occurs in up to 31% of patients in some series^8^.

The prophylactic placement of a mesh has been studied with the aim of reducing the incidence of aponeurosis dehiscence. Its use in the pre-aponeurotic position avoids aponeurosis dehiscence and, consequently, evisceration, eventration, and incisional hernia in high-risk patients undergoing median laparotomy in conditions such as emergency^9^, bariatric surgery^9^ ^-^ ^12^, elective abdominal aortic aneurysm repair^13^ ^-^ ^15^, and colorectal surgery^16^ ^,^ ^17^. Regardless of the indication, other studies have demonstrated the benefit of the prophylactic mesh in patients at high risk for incisional hernia and aponeurosis dehiscence, even in the presence of peritonitis^18^ ^-3^.

Thus, the objective of the present study is to evaluate the characteristics of a series of patients submitted to resuture of the abdominal wall due to evisceration and to verify whether the placement of a prophylactic pre-aponeurotic mesh, with an indication based on the scale developed by Lima et al.^9^, adapted from the studies by Ramshorst et al.^9^ ^,^ ^24^ and Goméz Diaz et al.^25^, could bring benefits this population.

## METHODS

This is a retrospective cohort study that analyzed patients who underwent abdominal wall resuture between January 1, 2010 and December 31, 2023 in a tertiary hospital in Southern Santa Catarina, Brazil. We analyzed patients medical records and tabulated the variables of interest, including data regarding epidemiological and laboratory profiles, and type and technique of closure of the first surgery. In the Service, sutures usually used to close the midline aponeurosis are polygalactin 1 or double nylon 0, according to the surgeon’s preference. There was no technical standardization in this cohort, such as the currently widespread small-bites technique.

The inclusion criteria were patients who underwent abdominal wall resuture at the study hospital, with first surgery at the same institution, median access, and age over 18 years. The exclusion criteria were patients younger than 18 years of age, with non-median accesses, or undergoing laparoscopic surgery. The institution’s Ethics Committee approved the study through Opinion CAAE 80030824.4.0000.5364. We followed the STROBE^26^ guidelines for the preparation of the final text. 

**Figure 1:**
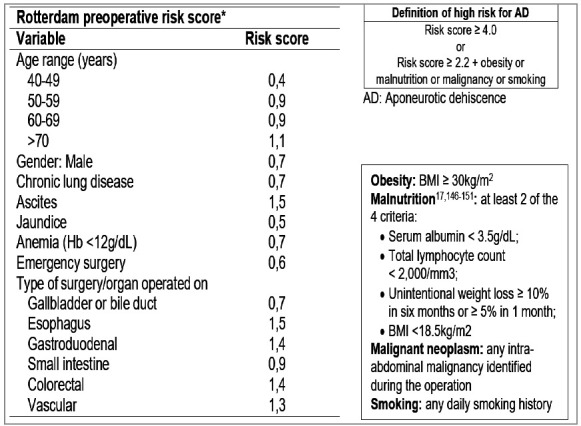
Rotterdam score, adapted by Lima et al., and definitions of high risk.

Figure 1, extracted from Lima et al.^9^, was used to calculate the Rotterdam score and brings the definitions of high risk for aponeurosis dehiscence.

Statistical analysis was performed by constructing pivot tables in Microsoft Excel 2016 and analysis in IBM SPSS v27. We used the Shapiro-Wilk test to assess data normality, with median and interquartile range measures being used to represent non-normal distributions and mean and standard deviation measures for normal ones. We used the Kruskal-Wallis test for correlations between nominal and scalar data. We correlated scalar data with each other with the Chi-square test, with correction by the Spearman’s test.

## RESULTS

During the study period, 436 patients underwent abdominal wall resuturing. Of these, 252 met the inclusion and exclusion criteria, thus resulting in the final sample. Most patients were male. The median age was 64 years (53-73) and the median Body Mass Index (BMI) was 24.3 kg/m^2^ (21.2-26.8).

There was no statistically significant correlation between the patients‘ age and the time in days to evisceration by the Spearman’s test (p=0.752), nor between BMI and days to evisceration (p=0.927) by the same test. Table 1 shows the characteristics of the study population.

**Table 1:** Characteristics of the population.

Variable		n
Sex	Male	187 (74,2%)
	Female	65 (25,8%)
Age		64 (53-73)
BMI range		
	<18,5	15 (6,0%)
	18,5-24,9	101 (40,1%)
	25,0-29,9	61 (24,2%)
	30,0-34,9	21 (8,3%)
	35,0-39,9	2 (0,8%)
	≥40,0	0 (0,0%)
	Not informed	52 (20,6%)
Days between first surgery and resuture surgery		8,0 (5,0-12,0)
Need for a third approach?		25 (10,1%)
Preoperative albumin		
	Not collected	197 (80,1%)
	<2,5	26 (10,6%)
	2,5-3,5	15 (6,1%)
	>3,5	7 (2,8%)
Neutrophil/lymphocyte ratio		7,5 (4,2-12,5)
Hemoglobin in g/dL		11,1 (9,6-13,0)
Neoplasm		
	No	131 (52,4%)
	Yes	118 (47,2%)
Type of neoplasm		
	Colorectal	44 (36,9%)
	Gastric	17 (14,3%)
	Esophageal	15 (12,6%)
	Hepatopancreatobiliary	12 (10,3%)
	Urologic	11 (9,5%)
	Gynecologic	6 (5,0%)
	Other	13 (10,9%)
Smoking		
	No	167 (67,1%)
	Yes	80 (32,1%)
Ascites		
	No	238 (95,9%)
	Yes	8 (3,2%)
Variable		n
Total bilirubin*		1,48 ± 2,34
COPD		
	No	214 (85,9%)
	Yes	34 (13,6%)

Nominal variables represented in absolute number and percentage. Percentage values based on non-omitted cases. * Total bilirubin values represented as mean and standard deviation. Other scalar variables had a non-normal distribution and are represented in median and interquartile range. BMI: Body Mass Index, in kg/m^2^. COPD: Chronic Obstructive Pulmonary Disease.

Table 2 shows the characteristics of the procedures performed. There was no significant difference when comparing the types of sutures used in aponeurosis closure with the time in days until evisceration by the Kruskal-Wallis test (p=0.05).

**Table 2:** Characteristics of the procedure.

Variable		n
Character of the procedure		
	Elective surgery	146 (58,8%)
	Emergency surgery	101 (40,7%)
Type of procedure		
	Colorectal	86 (34,4%)
	Small intestine	40 (12,0%)
	Surgical feeding route	27 (10,8%)
	Gastrorrhaphy	15 (6,0%)
	Appendectomy	12 (4,8%)
	Trauma laparotomy	12 (4,8%)
	Multiple Biopsies - Inoperable Tumor	10 (4,0%)
	Explorer Laparotomy	9 (3,2%)
	Urologic	8 (3,2%)
	Gastrectomy	7 (2,8%)
	Laparotomy for acute inflammatory abdomen	5 (2,0%)
	Pancreaticodenogastroduodenectomy	4 (1,6%)
	Hysterectomy/Oophorectomy	3 (1,2%)
	Abscess Drainage	3 (1,2%)
	Biliodigestive Diversion	2 (0,8%)
	Splenectomy	2 (0,8%)
	Gastroenteroanastomosis	2 (0,8%)
	Hepatectomy	1 (0,4%)
Suture Type		
	Double Nylon 0	137 (54,4%)
	Polyglactin 1	73 (29,0%)
	Other/Not informed	42 (16,6%)
Drain Use		
	No	175 (71,4%)
	Yes	70 (28,5%)

Nominal variables represented in absolute number and percentage. Percentage values based on non-omitted cases.

Table 3 shows the the Rotterdam score, with the risk of aponeurosis dehiscence for each interval, as well as the number of patients in each risk group. It can be seen from the right column that many patients, despite showing relatively low scores, have risk factors that, as shown in Figure 1, add up to a greater chance of aponeurosis dehiscence.

**Table 3:** Grouping of patients according to the Rotterdam score.

Sum	Risk of AD	N (%)	N with risk factors
0 - 2	0,1%	16 (7,0%)	11
2 - 4	0,7%	179 (78,8%)	137
4 - 6	5,5%	32 (14,1%)	29
6 - 8	26,2%	-	
> 8	66,5%	-	

AD: Aponeurotic Dehiscence. The columns “Sum” and “Risk of AD” are related to the Rotterdam score. The columns “N (%)” and “N with risk factors” refer to the patients in this study. Percentage values based on non-omitted cases. “Risk factor” includes patients with obesity, malnutrition, malignant neoplasm, and smoking (Figure 1).

In Table 4, the patients are grouped according to the indication of prophylactic mesh, based on the Rotterdam criteria modified by Lima^9^. Of the 227 patients with sufficient data to be grouped by the score, 164 patients, corresponding to 72.2% of those classifiable, would have received prophylactic mesh, which would potentially have prevented evisceration. These patients are those with a score greater than 2.2 and a risk factor or those with a score greater than 4.0 without a risk factor.

**Table 4:** Grouping of patients according to prophylactic mesh indication.

Score	n	Score	n
<2.2 with RF	16	<4.0 without RF	47
2.2-3.9 with RF	132	≥4.0 without RF	3
≥4.0 with RF	29	Insufficient data	26

RF: Risk factor. See Figure 1 for definitions.

## DISCUSSION

The higher rate of males among those eviscerated has already been demonstrated in other studies. In the series by Lima et al., the male/female ratio was 6:1. Other studies also show more than 70% of the eviscerated patients being male^9^ ^,^ ^24^ ^,^ ^27^. The age in this series was also similar to that found in the literature, which ranges from 65 to 70.6 years^24^ ^,^ ^9^.

Regarding BMI, in the present study 23 patients were obese, defined as BMI greater than 30kg/m^2^, which corresponds to 11.5% of the patients with this data in the medical records. In the study by Mir et al.^27^, 54.5% of patients had a BMI greater than 27kg/m^2^, and another series found obesity in 29.7% of the participants^6^. Argudo used a BMI greater than 29kg/m^2^ in his score to indicate the use of mesh^17^, suggesting this variable as an important risk factor for evisceration. 

The median time between the day of surgery and the date of resuture was 8 (5-12) days, similar to that found in other studies^6^ ^,^ ^9^. Regarding preoperative albumin, only 48 medical records had this data. Even so, 16.7% of the total patients had serum albumin lower than 3.5 g/dL. The Mir study, similarly, showed that 23.6% of patients with aponeurosis dehiscence had an albumin value lower than 3g/dL^27^. 

Anemia is a risk factor with a negative weight in wound healing^28^ ^,^ ^29^. The median hemoglobin found in this sample was 11.1 g/dL (9.6-13.0), which means that 50% of the sample had a hemoglobin value below 11.1 g/dL and 25%, below 9.6 g/dL. Anemia had a prevalence of 61% in the Van Ramshorst sample^24^ and 62.1% in the Mir series^27^.

The concomitance of neoplasia in patients who underwent resuture was 47.2%, similar to that demonstrated in other studies, in which it ranged from 42% to 48.6%^6^ ^,^ ^24^ ^,^ ^27^. On the other hand, the proportion of smokers, in this case 32.1%, fluctuated more widely in similar studies, from 46.4%^27^ to 72.0%^6^.

Just as smoking is a known risk factor for surgical complications, the concurrency of chronic obstructive pulmonary disease - COPD - is a risk factor directly implicated in surgical wound dehiscence. In the present study, 13% of the patients had COPD, whereas in the literature this rate is higher, at 29%^24^ and 49%^6^. Similarly, ascites, present in 3.2% of this sample, was found in 23%^24^ to 24.3%^27^ in other studies. 

An important point concerns the elective or urgent nature of the procedures performed. In the study by Lima^9^, all patients were operated on an urgent basis, unlike our series, in which only 40.7% of the eviscerated patients came from this scenario. Other studies, however, obtained a similar sample, with 42.9%^27^ to 46%^24^ of patients eviscerated secondary to emergency surgeries. 

Regarding the type of suture used in the aponeurosis closure, we found no significant difference when comparing the types of sutures used with the time in days until evisceration. This may be explained by the thesis that because evisceration is an earlier event, the type of material thus having less influence when compared to incisional hernia^24^. Long-term follow-up studies are needed for this confirmation.

Studies have already shown a reduction in the incidence of aponeurosis dehiscence and incisional hernia with the use of pre-aponeurotic prophylactic mesh, generally with a high-weight macroporous mesh^10^ ^,^ ^17^ ^,^ ^19^ ^,^ ^30^, with incisional hernia rates of 20% for control groups and 0% in the preaponeurotic mesh group, rendering an Number Needed to Treat (NNT) of 5^13^. Lima et al.^9^ allocated 52 patients to the suture group and 63 to the prophylactic mesh group, and aponeurosis dehiscence occurred in 13.5% in the first group and none in the second, therefore with an NNT of 7.4. 

Some limitations are evident in this series, especially the retrospective, unicentric nature and the small number of patients who actually presented evisceration. Prospective studies have certainly elucidated important points on the subject. It is interesting to note that the routine of the service under study recommends the use of polyglactin or nylon threads to close the aponeurosis. The use of polydioxanone threads has been increasingly used and accepted in the literature. This difference, in the long term, may have some impact on complications such as chronic pain and incisional hernias, a fact that will still be studied. 

It was well demonstrated, however, that with the application of the Rotterdam score modified by Lima et al.^9^, especially due to the valuation of risk factors, 72.2% of patients who presented aponeurosis dehiscence could be spared a surgical reapproach for abdominal wall resuture if the use of prophylactic mesh in the first surgery was chosen, which is the main finding of this study. 

There is no way to exhaust the theme without the use of the most effective techniques for closing the aponeurosis. The small-bites technique is recommended in the most recent guidelines, despite the still low level of evidence^31^ ^,^ ^32^. Thus, prospective studies should explore this technique, including evaluating the rate of aponeurosis dehiscence and the possible modifications in the NNT in favor of the prophylactic mesh, to verify the real relevance of this promising technique.

## CONCLUSION

The use of prophylactic mesh in the patients of the present series, through the application of the risk score for dehiscence of the Rotterdam aponeurosis modified by Lima et al.^9^, would have potentially prevented evisceration in 72.2% of patients.
